# No genetic causal association between periodontitis and ankylosing spondylitis: a bidirectional two-sample mendelian randomization analysis

**DOI:** 10.1186/s12920-024-01845-3

**Published:** 2024-05-02

**Authors:** Chong Han, Dongchao Wu, Feiyan Yu, Qianqian Wang, Yang Yang, Yi Li, Rao Qin, Yue Chen, Linkun Xu, Dongning He

**Affiliations:** 1 Shanxi Medical University School and Hospital of Stomatology, Taiyuan, China; 2 Shanxi Province Key Laboratory of Oral Diseases Prevention and New Materials, Taiyuan, China; 3 Department of Implantology, Shanxi Medical University School and Hospital of Stomatology, No. 63, New South Road, Yingze District, 030001 Taiyuan, Shanxi P.R. China

**Keywords:** Periodontitis, Ankylosing spondylitis, Mendelian randomization, Causal relationship

## Abstract

**Background:**

Observational studies that reveal an association between periodontitis (PD) and ankylosing spondylitis (AS) exist. However, observational research is prone to reverse causality and confounding factors, which make it challenging to infer cause-and-effect relationships. We conducted a two-sample Mendelian randomization (MR) study to examine the causal relationship between the genetic prediction of PD and AS.

**Methods:**

In our study, single-nucleotide polymorphisms (SNPs) were defined as instrumental variables (IVs). The genetic association with PD came from the Gene-Lifestyle Interactions and Dental Endpoints (GLIDE) consortium, wherein 17353 cases of European ancestry and 28210 controls of European ancestry were included in this study. The genetic association with AS from the Neale Laboratory Consortium included 337,159 individuals from the United Kingdom, with 968 cases and 336,191 controls. MR analysis was mainly performed using the inverse-variance weighted (IVW) method. In addition, the robustness of the study findings was assessed using sensitivity, pleiotropy, and heterogeneity analyses.

**Results:**

Eighteen independent SNPs with *P*-values significantly smaller than 1 × 10^− 5^ were used as IV SNPs for PD, while 39 independent SNPs with *P*-values significantly smaller than 1 × 10^− 5^ were used as IV SNPs for AS. The results of the IVW method revealed no causal association between PD and AS (odds ratio = 1.00, 95% confidence interval: 0.99953 to 1.00067, *P* = 0.72). The MR-Egger method did not support the causal association between PD and AS. It is unlikely that horizontal pleiotropy distorts causal estimates based on sensitivity analysis. No significant heterogeneity was observed in the Q test. The ‘’leave-one-out’’ analysis demonstrated that the robustness of our results was unaffected by eliminating any of the IVs. Likewise, no significant causative effect for AS on PD was observed in the inverse MR analysis.

**Conclusions:**

The study results do not support shared heritability or a causal association between PD and AS.

**Supplementary Information:**

The online version contains supplementary material available at 10.1186/s12920-024-01845-3.

## Introduction

Periodontitis (PD) is a chronic inflammatory disease affecting teeth-supporting tissues and is a multifactorial disease wherein genetic and environmental factors play a crucial role in its development [1]. Dental plaque initiates the development of PD; however, it is the body’s inflammatory response caused by a bacterial invasion that promotes immune cell-mediated self-degradation of periodontal tissues, resulting in tooth loss [2]. This suggests that immune function alterations might largely influence PD progression. Notably, most of an organism’s adaptive immune features are influenced by genetic factors, and some complex immune features set in on exposure to specific pathogens [3]. In recent years, several epidemiological studies have confirmed the association between immune-mediated rheumatic diseases and PD.

Ankylosing spondylitis (AS) is an immune-mediated chronic inflammatory rheumatic disease primarily affecting the axial skeleton [4]. The balance of the innate and acquired immune system is disrupted in patients with AS, resulting in soft and hard connective tissue destruction, causing inflammatory back pain [5]. The exact aetiology of AS is currently unknown; however, it has been reported to be associated with single nucleotide polymorphisms (SNPs) in genes encoding cytokines, which could interfere with cytokine production and promote the development of AS [6].

Attempts have been made by several epidemiological studies to propose an association between AS and PD. For example, a case-control study by Pischon et al. [7] reported a significantly higher incidence of PD in patients with AS compared with non-AS patients. In addition, a meta-analysis comprising six case-control studies reported a significantly higher incidence of PD in patients with AS [8]. However, a study by Kang et al. [9] reported no significant relationship between the disease duration and PD in patients with AS. Due to certain limitations of observational studies, such as their tendency to reverse causality, evidence on whether there is a causal relationship between AS and PD remains lacking.

In general, the gold standard for elucidating causality is a randomised controlled trial (RCT), wherein participants are randomly divided into control and experimental groups to study a particular factor’s effect [10]. However, the design and implementation conditions of RCTs are demanding, tightly controlled and challenging, and medical ethical issues must be considered. Therefore, relevant RCT studies have not been conducted. Mendelian randomization (MR), which draws on the idea of the instrumental variables (IVs) approach in economics, skilfully deals with the concern of interference in causal inference while using the summary data from genome-wide association studies (GWAS), effectively circumvents RCT study limitations and is extensively used in the field of PD [11–15]. The two-sample MR approach, wherein genetic variation in screening exposure and outcome is obtained from two independent samples from the same population source, further improves statistical efficacy by avoiding false-positive results of single-sample MRs to a certain degree [16].

This study examined the genetic causal relationship between AS and PD based on a large GWAS dataset within the framework of a two-sample MR analysis and provided a rationale for whether treating AS was necessary for patients with PD and whether dentists need to work closely with rheumatologists for managing PD.

### Overall study design

**Fig. 1:**
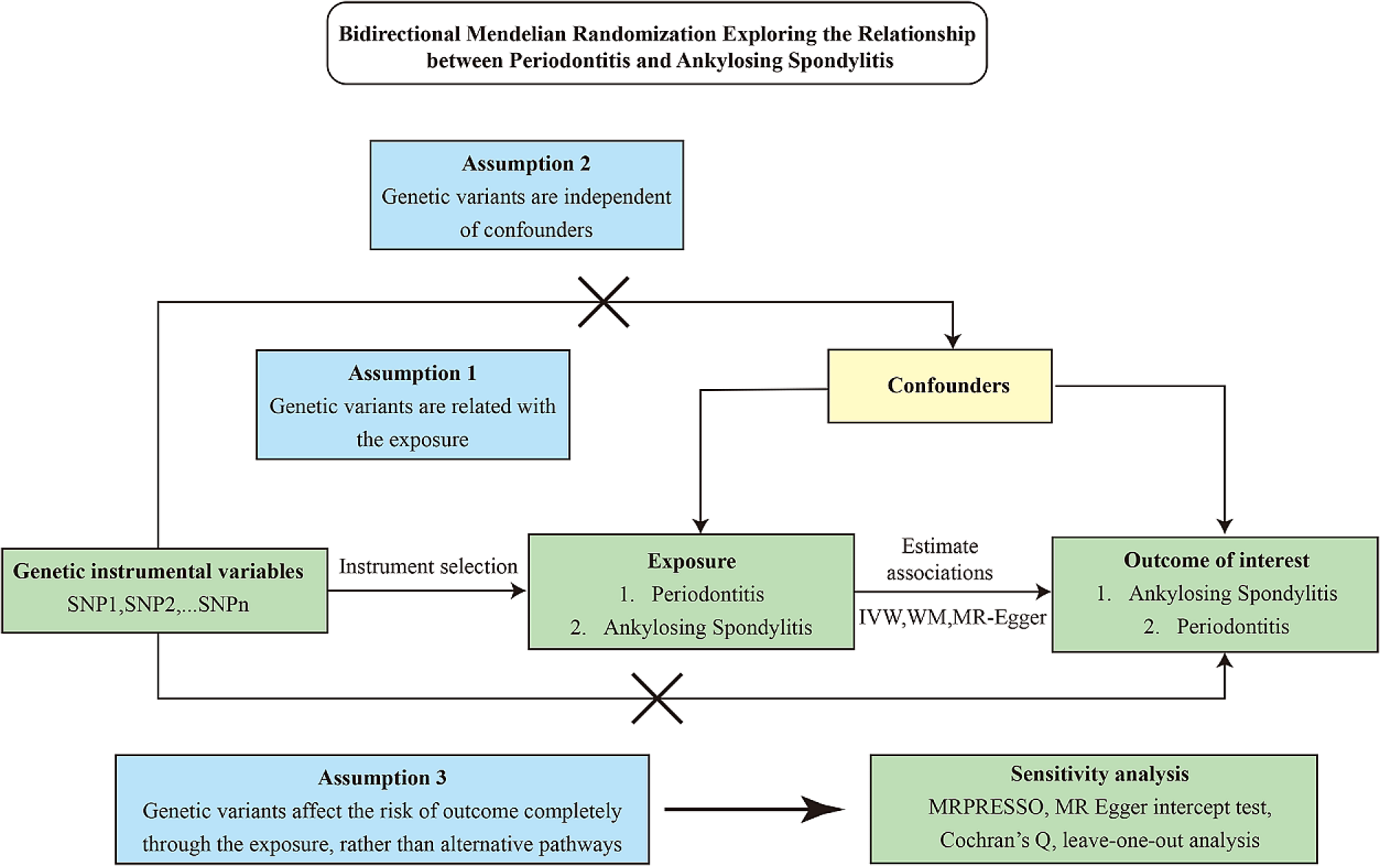
Schematics for the bidirectional Mendelian randomization design. Mendelian randomization requires valid genetic instrumental variants satisfying three assumptions

Our two-way two-sample MR study was conducted within a framework (Fig. 1). The causal effect and reverse causality of AS on PD were individually investigated. Herein, we used MR to investigate the causality of PD with AS. MR studies use genetic variation as an IV, and a valid IV must satisfy the following three assumptions [17]: (1) it must be strongly associated with exposure (“relevance”); (2) it must be independent of confounding factors for observational associations (“exchangeability”); (3) it must be associated with the outcome through exposure only (“exclusion restriction”).

### Data sources

Summary statistics for PD are provided in the latest meta-analysis from GWAS. The study included 17,353 cases of European ancestry and 28,210 controls of European ancestry from the Gene-Lifestyle Interactions and Dental Endpoints consortium [18]. The genetic association with AS came from the Neale Laboratory Consortium, wherein 337,159 individuals from the United Kingdom, with 968 cases and 336,191 controls, were included [19].

### Genetic instrument selection

A set of high-quality control steps were performed to select SNPs eligible from the GWAS summary results. The TwoSampleMR package (0.5.6) from R software(version 4.1.0) [20] was used to perform the analysis, which provided a simplified method of running MR analysis using summary-level data. This two-sample MR analysis considers genome-wide significant (*P* < 1 × 10^− 5^) SNPs as the IV to estimate the causal effect of PD and AS. A clustering procedure (R^2^ < 0.001 and window size = 10,000 kb) was performed for individuals of European origin in the 1000 Genomes Project to rule out SNPs with strong linkage disequilibrium (LD) [21]. Additionally, we calculated the F-statistics and extracted the sensitive SNPs based on F-statistics ≥ 10 for further analysis. Finally, the PhenoScanner database (www.phenoscanner.medschl.cam.ac.uk/) was used to search for previously reported associations of instrumental SNPs (and LD proxies) with potential confounders and selected thresholds (*P* < 1 × 10^− 5^, R^2^ > 0.8) to enhance the instrumental analysis.

### Statistical analysis

Herein, the instrument’s strength was explained using the pseudo-R^2^ and F-statistics of the responsible variants’ proportion for the SNPs. We performed a two-sample MR with multi-target GWAS for individual query requests for the selected SNPs, coordinated the effect alleles in each study, trimmed the LD, and performed sensitivity analyses.

We used the inverse-variance weighted (IVW) method for calculating MR estimates to assess bidirectional causality, supplemented by weighted median and MR-Egger methods that are relatively robust to horizontal pleiotropy. We considered a causal relationship between PD and AS when a one-tailed *P* < 0.05. Primary analysis is performed using the IVW method, which calculates a weighted average of the Wald ratio estimates. The IVW method provides the most accurate results when all selected SNPs are valid for the IVs [22]. A weighted linear regression is performed by the MR-Egger regression under the assumption that the instrumental strength is independent of direct effects (InSIDE) and produces coherent causal estimates, although not all genetic IVs are valid [23].

The weighted median regression method calculates the weighted median of the Wald ratio estimates, is robust to horizontal pleiotropic bias and is independent of the InSIDE assumption [24]. The weighted median method proved to be advantageous over the MR-Egger regression method, as it lowered type I errors and increased the causal estimation power [25].

### Pleiotropy and sensitivity analysis

The MR-Egger intercept test was used to assess the horizontal pleiotropy of significant SNPs, in which a one-tailed *P* < 0.05 indicates horizontal pleiotropy. Meanwhile, we used the Q statistic in this study to assess the heterogeneity among SNPs in the IVW method estimates. A one-tailed *P* value of < 0.05 in the Q statistic was considered significant heterogenicity. In addition, the MR Pleiotropy REsidual Sum and Outlier (MR-PRESSO) approach was used to assess the presence of pleiotropy, where a one-tailed *P* < 0.05 indicated statistically significant. The horizontal pleiotropy can be corrected via the removal of outliers and determine if the causal effect before and after the removal of outliers has changed substantially. Funnel plots and scatter plots of MR analysis can be used to visually assess horizontal pleiotropy and heterogeneity.

## Results

### Causal effects of AS on PD

Thirty-nine independent SNPs with significant *P*-values of < 1 × 10^− 5^ were used as IV SNPs for AS. These 39 IV SNPs could be found in the summary statistics of PD and were not significant in PD expression. These SNPs were not palindromic SNPs with moderate allele frequencies. These SNPs were uploaded to the PhenoScanner database, and we eliminated two SNPs significantly associated with rheumatoid arthritis (rs3130649 and rs75502439), one SNP significantly associated with the body mass index (BMI) (rs58742490), and one SNP significantly associated with smoking SNPs (rs6456815), and ultimately 35 SNPs were screened for subsequent MR analysis. Detailed information on the IVs for AS is presented in Supplementary Table S1.

**Fig. 2:**
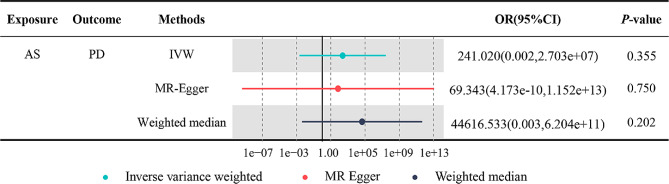
Comparisons of Mendelian randomization results by different methods. CI: confidence interval; IVW: inverse variance weighted; MR: Mendelian randomization; OR: odds ratio; AS: ankylosing spondylitis; PD: periodontitis

**Fig. 3:**
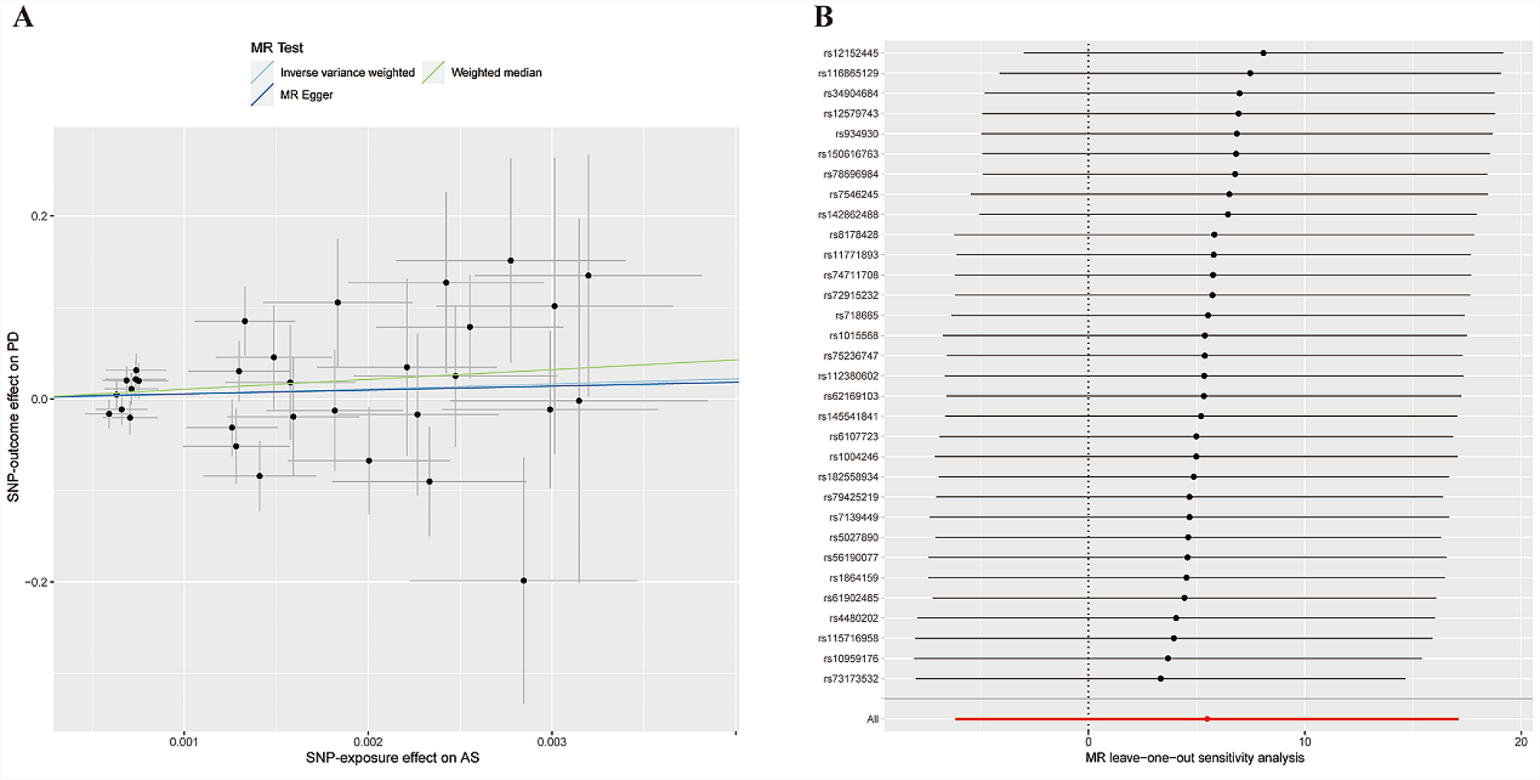
MR analysis and leave-one-out analysis of the causal effect of AS on PD susceptibility. (**A**) A scatter plot depicting the causal relationships between AS and PD using various MR methods. Each line’s slope corresponds to the expected MR effect for each method. (**B**) The causal links between AS and PD are depicted using a leave-one-out plot. The leave-one-out figure depicted how the removal of a single variant altered the causal estimations (point with horizontal line) for the effect of AS on PD

We used the IVW method supplemented with methods that have more stability for horizontal pleiotropy, including weighted median and MR-Egger methods, to calculate the MR estimates. The IVW method revealed no significant relationship between an increased risk of developing AS and an increased risk of developing PD (AS: odds ratio [OR] = 241, 95% confidence interval [CI]: -6.14 to 17.11, *P* = 0.36) and the same results were obtained for the MR-Egger and weighted median methods (Table 1; Fig. 2). Therefore, we can assume that AS is not causally related to PD (Fig. 3).

**Table 1:** Estimates the causal effects between PD and AS through MR methods

Exposure	Outcome	Methods	N.SNP	OR	95%CI	P-value
AS	PD	IVW	39	241.020	(0.002,2.703E + 07)	0.36
		MR- Egger	39	69.343	(4.173e-10,1.152E + 13)	0.75
		WM	39	44616.533	(0.003,6.204E + 11)	0.20
PD	AS	IVW	18	1.0001	(0.9995,1.0007)	0.72
		MR- Egger	18	1.0001	(0.9993,1.0009)	0.77
		WM	18	1.0005	(0.9996,1.0013)	0.27

*Abbreviations* AS: Ankylosing Spondylitis; PD: Periodontitis; CI: confidence interval; IVW: inverse variance weighted; MR: Mendelian randomization; WM: Weighten median; N.SNPs: number of SNPs used in MR; OR: odds ratio

No heterogeneity was observed between individual SNPs based on the heterogeneity test. The MR- Egger regression analysis revealed that no potential pleiotropic effect exists for the variants used in constructing the genetic instruments (*P* = 38.55) (Table 2). The leave-one-out analysis revealed that eliminating any of the SNPs did not affect the causal estimates of AS (Fig. 3).

**Table 2:** The heterogeneity and horizontal pleiotropy of individual SNPs

		Heterogeneity					
		MR-Egger			IVW		
Exposure	Outcome	Q	df	P-value	Q	df	P-value
AS	PD	38.54	30	0.14	38.55	31	0.17
PD	AS	8.71	14	0.85	8.71	15	0.89
		**MR-Egger test for horizontal pleiotropy**					
**Exposure**	**Outcome**	**Intercept**		**SE**		***P*** **-value**	
AS	PD	0.0014		0.0128		0.9160	
PD	AS	-4.61E-06		7.46E-05		0.9517	

*Abbreviations* *df*: degree of freedom; MR: Mendelian randomization; *Q*: heterogeneity statistic *Q*

### Causal effects of PD on AS

Similarly, we used 18 independent SNPs with *P*-values significantly < 1 × 10^− 5^ as IV SNPs for PD. All 18 IV SNPs could be found in the summary statistics of AS and were not significant for AS expression. These SNPs were not palindromic SNPs with moderate allele frequencies. These SNPs were uploaded to the PhenoScanner database, and we eliminated one SNP associated with BMI (rs2921075). Ultimately 17 SNPs were screened for use in the subsequent MR analyses. Detailed information on the IVs for PD is presented in Supplementary Table S2.

The IVW, weighted median, and MR-Egger methods were used to calculate the MR estimates. Our main IVW method results revealed a positive correlation between PD and AS (Supplementary Figure S1); however, it was not statistically significant (PD: OR = 1.00, 95% CI: 0.99953 to 1.00067, *P* = 0.72). In addition, the MR-Egger and weighted median method results were consistent with the above-mentioned results (Supplementary Figure S2). In general, evidence supporting a causal relationship between PD and the development of AS was credible.

The heterogeneity test revealed that the individual SNPs were not heterogeneous. Based on the MR- Egger regression analysis results, it is no potential pleiotropic effect exists for the variants used in constructing the genetic instruments (*P* = 8.71) (Table 2). The leave-one-out analysis revealed that eliminating any of the SNPs does not affect the causal estimation of PD (Supplementary Figure S3).

## Discussion

To the best of our knowledge, this is the first study to validate the bidirectional causal relationship between PD and AS using MR methods in a population of European ancestry. Our MR study using large-scale GWAS data revealed no causal relationship between PD and AS and vice versa. The fact that there are few GWAS studies for both diseases is noteworthy, and these findings should be interpreted with caution.

Reportedly, patients with AS are at a higher risk of developing PD [7, 26–28]. Keller [26] reported that patients with AS were more likely to develop chronic PD than controls (OR = 1.84, 95% CI: 1.74 to 1.98). Pischon [7] reported that patients with AS were 6.81 times (95% CI: 1.96 to 23.67) more likely to develop PD (defined as a mean attachment loss of 0.3 mm) compared with the controls. A recent meta-analysis [8] reported that the prevalence of PD ranged from 38 to 88% in patients with AS and 26–71% in patients without AS, and patients with AS were at a significantly higher risk of developing PD (OR = 1.85, 95% CI: 1.72 to 1.98). However, not all studies have established an association between PD and AS. Sezer [29] reported no significant relationship between the disease duration in patients with AS and some of the diagnostic indicators of PD, such as probing depth and clinical attachment level (CAL). Furthermore, Agrawal et al. [30] reported no association between human leukocyte antigen B27 (a diagnostic indicator of AS) and probing depth or CAL. Similarly, in a case-control study, no significant differences were observed between the AS and control groups in terms of the probing depth and CAL [31]. KANG [9] reported that the prevalence of PD in the AS group and the control group were 70.2% versus 66.6%, with no statistically significant difference. It is worth noting that this study was conducted on Asian individuals but not European individuals, and it is difficult to determine whether the outcome was influenced by a population stratification effect. In conclusion, the results of the various observational studies are inconsistent and do not prove whether a relationship between AS and PD exists.

Several possible explanations exist for the association between AS and PD in observational studies. The most important association between AS and PD is between non-major histocompatibility complex genes, which can disrupt the cytokine network balance involved in both diseases. The pathogeneses of PD and AS involve cytokines (tumour necrosis factor [TNF] and interleukins [ILs]), T lymphocytes, etc. [32, 33]. The pathogenic effects of IL-6, IL-2, TNF-α, and T immune cells are found in PD and AS, and there is evidence that IL-6, IL-2, and TNF-α levels are significantly elevated in patients with AS and PD [34–36]. In addition, it has been suggested that the osteoimmune mechanism of AS might be associated with PD [31]. Sclerostin is an osteoclast-specific protein that has been proven to be a diagnostic biomarker of AS [37–39]. Studies on sclerostin levels in serum and gingival sulcus fluid have shown significantly lower sclerostin levels in patients with AS than in the controls [38–40]. Intriguingly, when patients with AS were further subdivided into the PD and control groups, the results showed that the gingival crevicular fluid sclerostin levels were significantly lower in the PD group than in the control group and that the local sclerostin produced in the periodontal environment might be influenced by the patient’s systemic condition [41]. These findings caused several scholars to link AS and PD.

Although the above studies suggest that there might be some association between the two diseases due to a common immune response, whether there is a causal relationship between the two diseases lacks evidence. Our MR study does not support a causal relationship between the two diseases. It is possible that the previously observed association between PD and AS is coincidental or confounded by confounding factors. For example, a meta-analysis reported a statistically significant association between PD and obesity in individuals with an obese BMI (OR = 1.81, 95% CI: 1.42 to 2.30) [42]. Additionally, a meta-analysis comprising 12 studies revealed an association between overweight/obese BMI and AS [43]. Therefore, BMI is a likely confounder between PD and AS. Herein, after ruling out BMI as a confounding factor, we observed no causal relationship between PD and AS genetically, further suggesting that the observational study results might be influenced by BMI. However, the specific pathogenesis needs to be elucidated by further relevant studies.

The current study has several advantages. First, the MR design lowers residual confounding and other biases, thereby strengthening causal inferences. This bidirectional MR analysis ensures that inferences of causal relationships between PD and AS are bidirectional. These relationships were evaluated in two independent populations, and the high degree of agreement increased the reliability of our study. Second, several sensitivity analyses were performed. Our confidence in the established associations was strengthened owing to the consistent estimates across models. Third, we applied the GWAS for PD and AS in a population of European descent to obtain sufficient statistical power and assess potential causal relationships between PD and AS, thereby minimising the effect of population stratification. The large sample size used for both outcomes proved to be more advantageous (ensuring that there is sufficient statistical power to detect even relatively weak causal effects). At the same time, the present study has several limitations. First, since there are few GWAS studies in other populations and the populations included in this study are of European origin, the generalisability of our results is debatable. We still need more data from non-European individuals and further studies to understand the relationship between PD and AS more deeply. Second, because the condition determination studies included in the Neale Laboratory Consortium followed clear norms and did not specifically classify AS (presence of comorbidities), GWAS studies on PD might not have identified consistent SNPs, which might have influenced the AS results to a certain degree. More summative GWAS data and additional genetic tools might be required to re-perform the MR analyses in the future.

In conclusion, by applying MR methods, our results support the absence of a causal effect of AS on PD, and vice versa. This result varies from that of previous observational studies and therefore provides new ideas for treating PD and AS. Whether dentists need to work closely with rheumatologists to manage patients with PD remains controversial.

### Electronic supplementary material

Below is the link to the electronic supplementary material.

Supplementary Material 1

## Data Availability

All data generated or analyzed during this study are included in this published article and its additional material. The periodontitis summary statistic data are available at https://data.bris.ac.uk/data/dataset/. The Ankylosing Spondylitis summary data are available at https://gwas.mrcieu.ac.uk/.
