# Supplementary material for: No genetic causal association between periodontitis and ankylosing spondylitis: a bidirectional two-sample mendelian randomization analysis
Source: BMC Med Genomics. 2024 May 2;17:118. doi: 10.1186/s12920-024-01845-3 (PMC11067206; doi:10.1186/s12920-024-01845-3)
Supplement: Supplementary file 1 — Supplementary Material 1 [file 12920_2024_1845_MOESM1_ESM.docx]

Supplementary Materials

**Table S1.** SNPs employed as instrument variables and their associations with exposure and outcome.

| SNP | Chr | Pos | EA/OA | EAF | Association with PD | | | |
| --- | --- | --- | --- | --- | --- | --- | --- | --- |
|  |  |  |  |  | *β* | S.E. | *P*-value | *F*-statistic |
| rs79425219 | 1 | 158372322 | A/C | 0.0153 | 0.002 | 0.0005 | 5.19E-06 | 0.060 |
| rs7546245 | 1 | 67750474 | C/T | 0.3236 | 0.001 | 0.0001 | 2.51E-06 | 0.064 |
| rs718665 | 1 | 186619852 | G/A | 0.9907 | -0.003 | 0.0007 | 7.04E-06 | 0.062 |
| rs75236747 | 1 | 2353972 | C/T | 0.0354 | 0.002 | 0.0004 | 7.84E-06 | 0.057 |
| rs78696984 | 2 | 13898616 | T/C | 0.0535 | 0.001 | 0.0003 | 9.33E-06 | 0.056 |
| rs150616763 | 2 | 103638129 | G/T | 0.0225 | 0.002 | 0.0004 | 5.24E-06 | 0.060 |
| rs62169103 | 2 | 120793626 | A/G | 0.0191 | 0.002 | 0.0005 | 6.23E-06 | 0.062 |
| rs72915232 | 2 | 199699037 | G/A | 0.0234 | 0.002 | 0.0004 | 2.58E-07 | 0.079 |
| rs190735015 | 2 | 147454763 | T/A | 0.0114 | 0.003 | 0.0007 | 7.46E-06 | 0.066 |
| rs5027890 | 3 | 62920293 | C/A | 0.9890 | -0.003 | 0.0006 | 8.52E-06 | 0.057 |
| rs12152445 | 3 | 3163269 | G/A | 0.0493 | 0.001 | 0.0003 | 4.42E-06 | 0.063 |
| rs145541841 | 3 | 66054984 | A/G | 0.0108 | 0.003 | 0.0006 | 2.84E-06 | 0.065 |
| rs182558934 | 3 | 154070633 | T/G | 0.0121 | 0.003 | 0.0006 | 2.20E-07 | 0.082 |
| rs34904684 | 5 | 118461560 | G/A | 0.2599 | -0.001 | 0.0001 | 2.16E-06 | 0.065 |
| rs138753323 | 6 | 32173599 | C/T | 0.0331 | 0.005 | 0.0004 | 4.86E-48 | 0.636 |
| rs115716958 | 6 | 17225712 | T/A | 0.0177 | 0.003 | 0.0005 | 5.21E-07 | 0.076 |
| rs114423170 | 6 | 30531413 | A/G | 0.0325 | 0.004 | 0.0004 | 2.16E-21 | 0.293 |
| rs116865129 | 6 | 100252020 | C/T | 0.0155 | 0.002 | 0.0005 | 9.76E-06 | 0.056 |
| rs1015568 | 6 | 16432284 | G/A | 0.5110 | -0.001 | 0.0001 | 1.53E-06 | 0.067 |
| rs11771893 | 7 | 137813718 | G/A | 0.0365 | 0.002 | 0.0004 | 9.06E-06 | 0.060 |
| rs74711708 | 8 | 10980215 | C/T | 0.0320 | 0.002 | 0.0004 | 8.92E-07 | 0.069 |
| rs4480202 | 9 | 38440859 | C/T | 0.4371 | -0.001 | 0.0001 | 1.86E-07 | 0.078 |
| rs10959176 | 9 | 10581485 | C/A | 0.1993 | 0.001 | 0.0002 | 6.65E-06 | 0.059 |
| rs61902485 | 11 | 59142038 | A/C | 0.0265 | 0.002 | 0.0004 | 6.20E-06 | 0.058 |
| rs12579743 | 12 | 115162946 | A/G | 0.0758 | 0.001 | 0.0002 | 4.20E-07 | 0.075 |
| rs7139449 | 12 | 80271069 | G/C | 0.9411 | -0.001 | 0.0003 | 2.72E-06 | 0.063 |
| rs934930 | 12 | 98985056 | C/T | 0.4813 | -0.001 | 0.0001 | 6.28E-06 | 0.059 |
| rs56190077 | 14 | 41295566 | G/A | 0.2083 | 0.001 | 0.0002 | 2.90E-06 | 0.063 |
| rs1864159 | 16 | 58011491 | G/A | 0.7782 | -0.001 | 0.0002 | 8.08E-06 | 0.063 |
| rs8178428 | 17 | 56347081 | T/C | 0.0136 | 0.003 | 0.0006 | 3.49E-07 | 0.081 |
| rs142862488 | 18 | 10188961 | T/A | 0.0121 | 0.003 | 0.0006 | 4.03E-06 | 0.065 |
| rs112380602 | 19 | 1176330 | T/C | 0.0166 | 0.002 | 0.0006 | 7.85E-06 | 0.067 |
| rs1004246 | 19 | 16456260 | T/C | 0.7310 | 0.001 | 0.0001 | 1.20E-06 | 0.067 |
| rs73173532 | 20 | 55549062 | T/C | 0.0605 | 0.001 | 0.0003 | 1.13E-06 | 0.068 |
| rs6107723 | 20 | 5931743 | C/T | 0.0446 | 0.001 | 0.0003 | 2.44E-06 | 0.064 |

Abbreviations: PD:periodontitis; AS: ankylosing spondylitis Chr: chromosome; EA/OA: effect allele/other allele; EAF: effect allele frequency; Pos: position according to GRCh37/hg19 genome assembly; SNP: single nucleotide polymorphism; SE: standard error of beta.

**Table S2.** SNPs employed as instrument variables and their associations with exposure and outcome.

| SNP | Chr | Pos | EA/OA | EAF | Association with AS | | | |
| --- | --- | --- | --- | --- | --- | --- | --- | --- |
|  |  |  |  |  | *β* | S.E. | *P*-value | *F*-statistic |
| rs35621881 | 1 | 49752432 | G/A | 0.1060 | 0.124 | 0.0277 | 7.85E-06 | 132.301 |
| rs151226594 | 11 | 88321793 | G/T | 0.0184 | 0.367 | 0.0768 | 1.75E-06 | 222.876 |
| rs138868497 | 11 | 77785437 | C/T | 0.0103 | -1.639 | 0.3324 | 8.20E-07 | 2638.854 |
| rs184267209 | 12 | 94396361 | A/G | 0.0266 | 0.246 | 0.0554 | 8.91E-06 | 143.229 |
| rs10143801 | 14 | 8305889 | G/A | 0.3258 | 0.084 | 0.0171 | 8.66E-07 | 141.667 |
| rs12438274 |  | 86508546 | G/T | 0.2290 | 0.088 | 0.0195 | 6.76E-06 | 124.078 |
| rs139182625 | 16 | 242925596 | T/C | 0.1397 | 0.102 | 0.0229 | 8.87E-06 | 113.775 |
| rs76734229 | 18 | 43560590 | A/G | 0.0737 | -0.176 | 0.0370 | 1.94E-06 | 193.733 |
| rs9954920 | 18 | 46460783 | T/C | 0.3572 | 0.077 | 0.0163 | 2.37E-06 | 124.064 |
| rs148287804 | 2 | 19970150 | G/T | 0.0167 | 0.383 | 0.0847 | 6.22E-06 | 220.441 |
| rs4811024 | 20 | 136207654 | G/C | 0.8945 | 0.134 | 0.0292 | 4.62E-06 | 154.236 |
| rs17071023 | 3 | 64256137 | G/C | 0.0204 | 0.267 | 0.0593 | 6.58E-06 | 130.577 |
| rs78422482 | 4 | 10854213 | A/G | 0.0310 | 0.219 | 0.0485 | 5.96E-06 | 132.142 |
| rs13220384 | 6 | 48909496 | C/A | 0.1230 | -0.108 | 0.0240 | 7.70E-06 | 114.088 |
| rs73155039 | 7 | 77555563 | G/A | 0.0106 | -0.832 | 0.1757 | 2.22E-06 | 670.619 |
| rs3779291 | 7 | 64539892 | C/T | 0.1481 | 0.098 | 0.0219 | 7.11E-06 | 111.134 |
| rs2921075 | 8 | 49752432 | C/G | 0.6927 | 0.092 | 0.0183 | 5.44E-07 | 163.334 |
| rs72712882 |  | 88321793 | A/G | 0.0926 | -0.131 | 0.0288 | 5.84E-06 | 130.968 |

Abbreviations: PD:periodontitis; AS: ankylosing spondylitis Chr: chromosome; EA/OA: effect allele/other allele; EAF: effect allele frequency; Pos: position according to GRCh37/hg19 genome assembly; SNP: single nucleotide polymorphism; SE: standard error of beta.


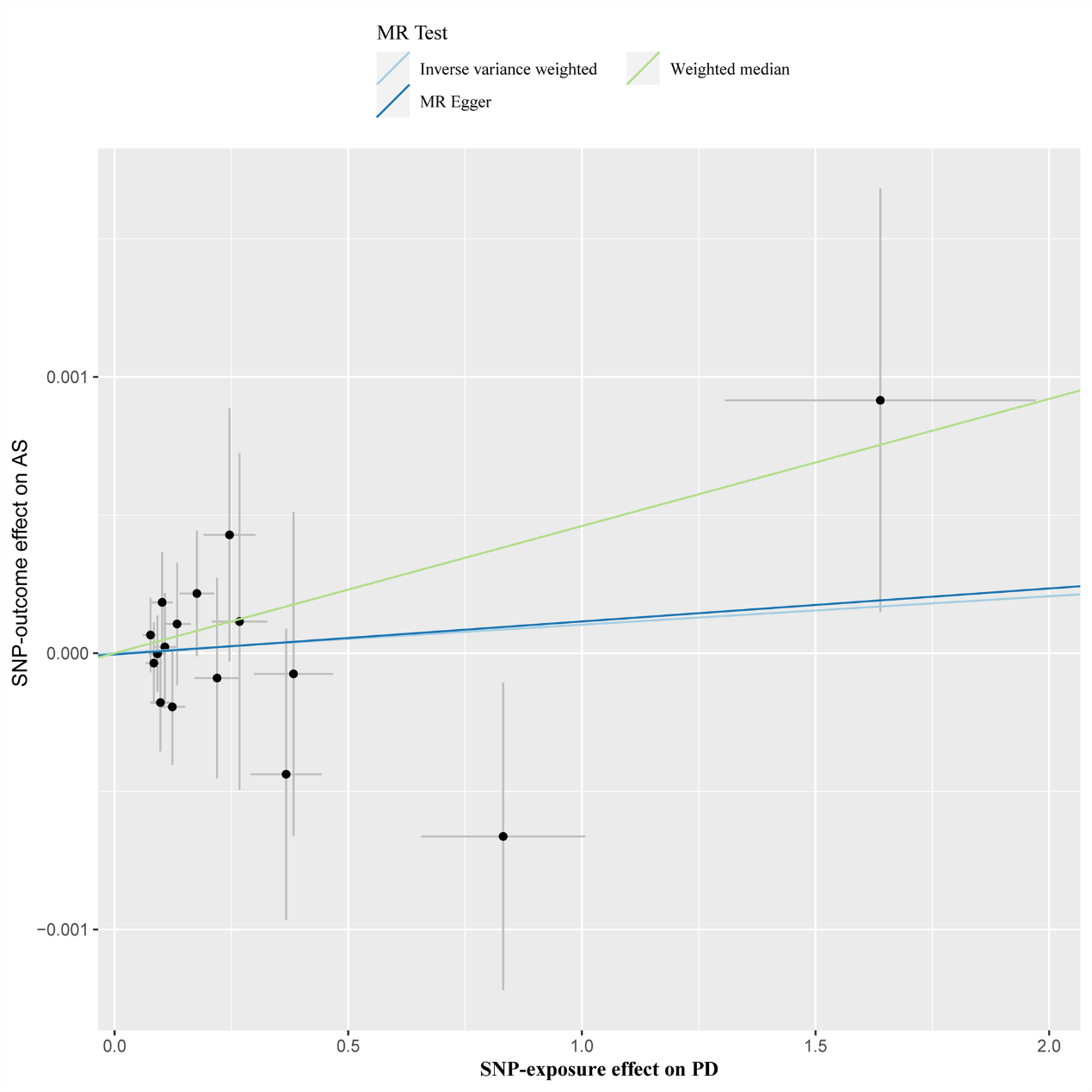


**Figure S1.** MR analysis of the causal effect of PD on AS susceptibility.A scatter plot depicting the causal relationships between PD and AS using various MR methods. Each line's slope corresponds to the expected MR effect for each method.


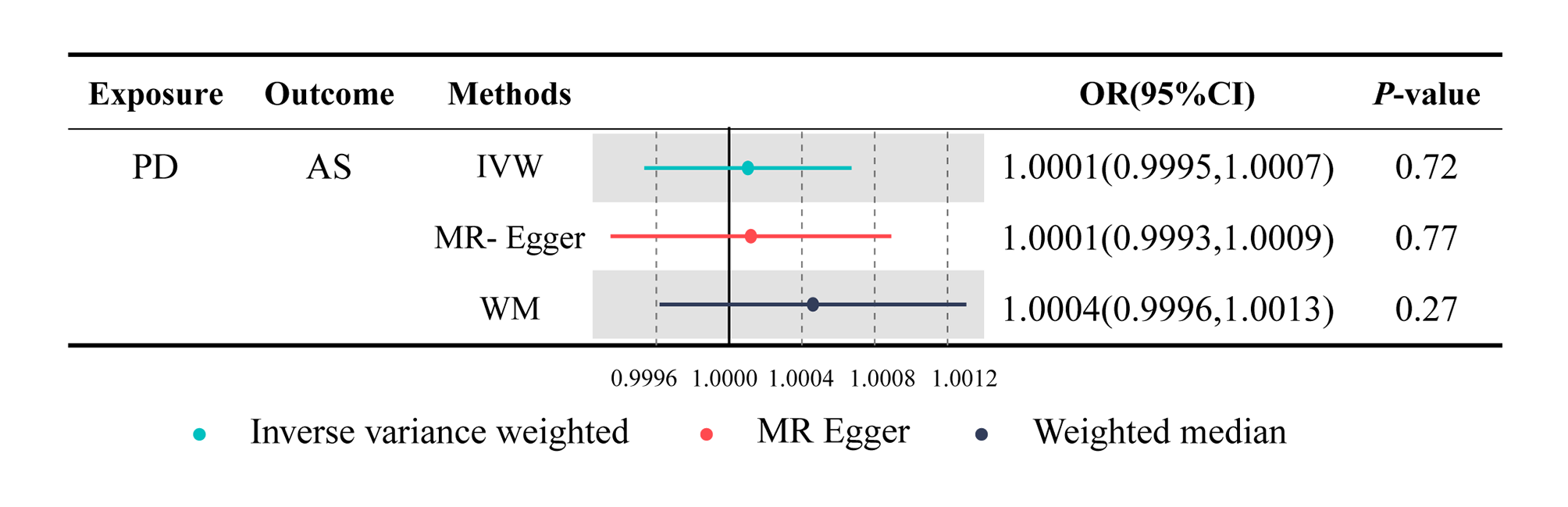


**Figure S2.** Comparisons of Mendelian randomization results by different methods. CI: confidence interval; IVW: inverse variance weighted; MR: Mendelian randomization; OR: odds ratio; PD: periodontitis; AS: ankylosing spondylitis.


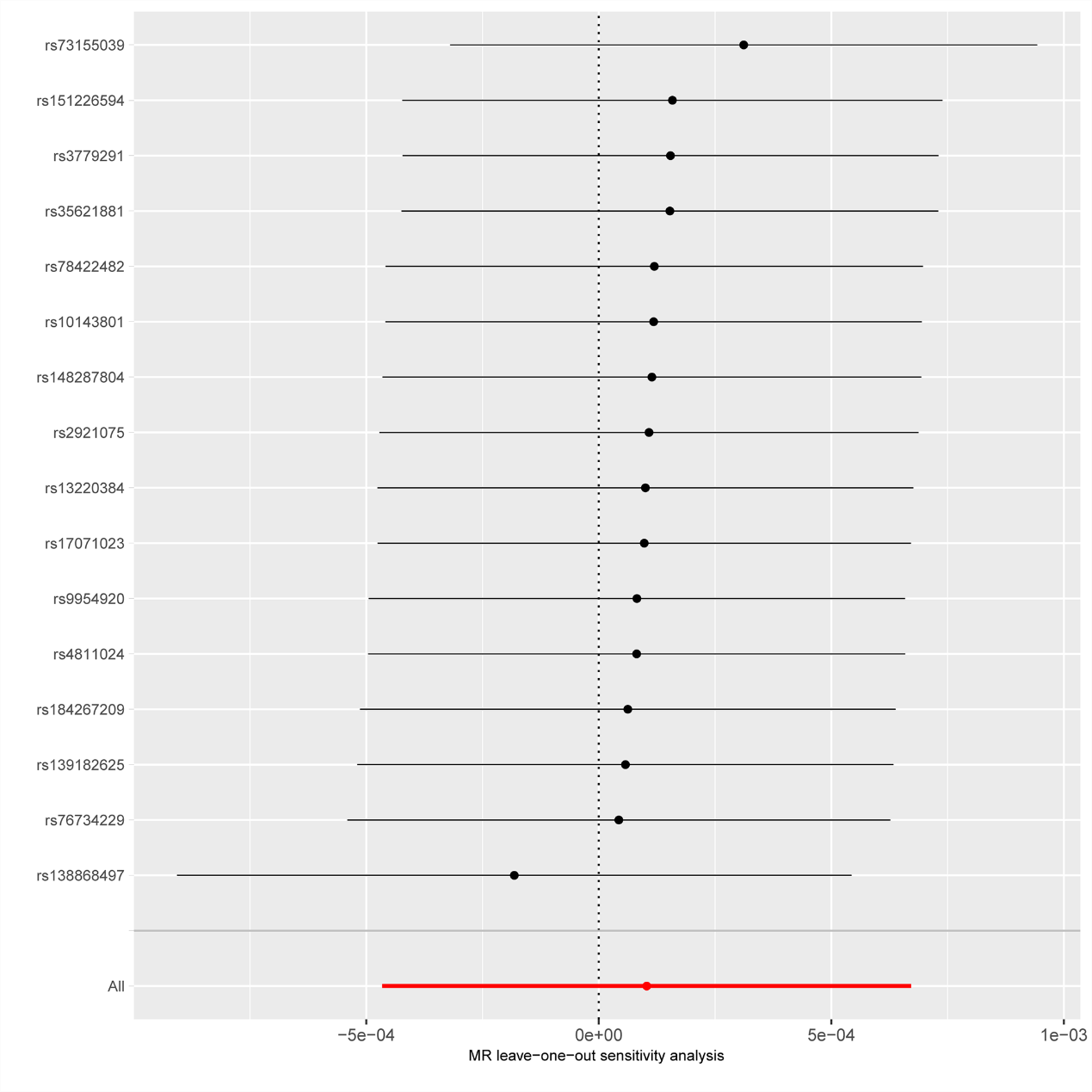


**Figure S3.** MR analysis and leave-one-out analysis of the causal effect of AS on PD susceptibility.The causal links between PD and AS are depicted using a leave-one-out plot. The leave-one-out figure depicted how the removal of a single variant altered the causal estimations (point with horizontal line) for the effect of PD on AS.
